# Impact of evolving cardiac catheterisation services on admissions to a regional ICU

**DOI:** 10.1186/cc14212

**Published:** 2015-03-16

**Authors:** EA Gorman, D Trainor

**Affiliations:** 1 Royal Victoria Hospital, Belfast, UK

## Introduction

National UK audit data demonstrate cardiac catheterisation services, including percutaneous coronary intervention and noncoronary interventions, are increasing [1-3]. National mortality rates post cardiac catheterisation are also increasing, reflecting an increasing proportion of sicker patients undergoing interventional procedures [3]. National audit procedures do not evaluate patients admitted to intensive care post cardiac catheterisation. We aimed to evaluate the impact of an evolving regional cardiac catheterisation service on a regional intensive care unit (RICU) serving a population of 1.8 million.

## Methods

A retrospective review was carried out. Patients admitted from the regional cardiac catheterisation laboratory to the regional ICU, between September 2009 and September 2014, were identified using validated RICU admission records. Clinical data were extracted from computerised patient records.

## Results

A total of 170 patients were identified (representing 2.9% of critical care admissions during this time). Baseline characteristics: 71.7% male, median age 66 (IQR 55 to 74), median APACHE score 18 (IQR 15 to 23). Seventy-one patients (41.7%) had an APACHE score >20. Fifteen patients (8.8%) were aged >80 years. Admissions increased yearly - 20 in 2010, 26 in 2011, 35 in 2012, 47 in 2013, 37 at the end of the third quarter of 2014 (projected 59 admissions by year end 2014). Median length of stay was 3.5 days (IQR 1.8 to 7.2). Average length of stay reduced yearly (9.14 days in 2010 to 5.01 days in 2014). ICU bed-days per year remained static over the 5-year period. Critical care and hospital mortality rates were 33% and 39% respectively. There was a trend towards increasing mortality yearly, and with increasing age and APACHE score.

## Conclusion

An evolving cardiac catheterisation service is having a significant impact on intensive care services within a regional centre. Increasing mortality trends in this critical care population reflects post-cardiac catheterisation mortality trends nationally. We suggest intensive care admissions post cardiac catheterisation should be included in the national audit, to allow forward planning of intensive care services and to promote quality improvement within this population.
